# Temporal variations in presynaptic release probability in the lateral habenula

**DOI:** 10.1038/srep40866

**Published:** 2017-01-20

**Authors:** Hoyong Park, Myunghyun Cheon, Sungmin Kim, ChiHye Chung

**Affiliations:** 1 Department of Biological Sciences, Konkuk University, 120 Neungdong-ro, Gwangjin-gu, Seoul 05029, South Korea

## Abstract

Rhythmicity plays an important role in a number of biological systems. The habenular complex is reported to contain an intrinsic molecular clock and to show rhythmic expression of circadian clock genes and proteins including per2/PER2. In this study, we observed that there is a temporal rhythmicity in the presynaptic efficacy of the lateral habenula (LHb) neurons. We collected a substantial number of recordings at different time points of the day during the light phase. The frequency and amplitude of spontaneous excitatory transmission were increased in the afternoon compared to recordings performed in the morning. In addition, the paired-pulse ratio and the success rate of minimal stimulation were also significantly different depending on the time of the recording. We did not see any significant differences in recordings obtained from pyramidal neurons of the hippocampus in the same brain slices. Taken together, our data indicates that the LHb exhibits intrinsic temporal oscillation in basal neurotransmission and in presynaptic release probability. Given the rapidly growing interest on the function of the LHb, more careful examination of synaptic transmission in the LHb is thus required.

Endogenous circadian rhythms mediate integrative responses of biological systems from individual cells to organs. The circadian rhythm in the brain is generated by the suprachiasmatic nuclei (SCN) and regulates hormonal and behavioural cycles^1^. Several previous studies have shown that SCN neurons express genes responsible for controlling circadian rhythm^2^, and control hormone concentrations such as those of corticosterone^3^,^4^ as well as sleep-awake cycles^5^. Recent studies have expanded the list of brain structures with circadian activities that can maintain circadian cycles *in vitro* for a while even in the absence of the SCN^6^. One such candidate is the lateral habenula (LHb), a small epithalamic brain region. In mammals, the retina detects and sends all light information through retinal ganglions cells (RGCs). Most of RGCs send light information to the visual cortex via the thalamus for visual perception. Melanopsin-containing retinal ganglion cells (mRGCs) are intrinsically photosensitive^7^ but contribute to non-image-forming vision, including the setting of the intrinsic circadian clock^8^,^9^. mRGCs are known to innervate brain areas other than the visual cortex including the hypothalamus, preoptic areas and the habenular complex in rodents^10^,^11^. Anatomically, the paraLHb is innervated by mRGCs, suggesting that the LHb receives multi-synaptic photo-sensitive inputs^12^,^13^. Anterograde tracing experiments have shown that the SCN innervates to the LHb^14^,^15^,^16^, although the innervations were quite sparse^14^,^16^ and not cross-confirmed by retrograde tracing experiments^17^. Retrograde tracing with pseudorabies virus has revealed that the LHb reciprocally projects to the SCN as well^18^. At the cellular level, LHb neurons are shown to express several clock proteins including Per1 and Per2^12^,^13^. Videomicroscopy imaging studies have revealed oscillatory bioluminescence responses in molecular clock proteins Per1 or Per2 fused with luciferase in the LHb^12^,^13^. Immunohistochemical experiments using hamsters have delineated that the pattern of c-fos expression in the LHb differs depending on the time of sacrifice for obtaining brain samples. The LHb obtained during the dark phase expressed more c-fos positive cells compared to that obtained during the light phase^19^. Functionally, some of LHb neurons were shown to be photically activated *in vivo*^12^,^20^. The spontaneous firing rates in the LHb but not in the medial habenula (mHb) have been reported to show circadian oscillations which lasted for at least two cycles in organotypic slice preparation^13^,^20^. However, rhythmicity in synaptic transmissions within the LHb has not been investigated so far.

Previously, we reported that miniature excitatory postsynaptic current (mEPSC) frequencies of LHb neurons projecting to the ventral tegmental area (VTA) are heterogeneous and exhibit a bimodal distribution from a large collection of recordings obtained throughout the day^21^. Here, we investigated whether the heterogeneity of neurotransmission in the LHb may rise from the intrinsic circadian oscillations in this structure and is under temporal control. We collected a substantial number of recordings of synaptic transmission in the LHb during restricted time windows of the day during the light phase. The heterogeneity of synaptic transmission maintained within the recording time; however we observed a very significant shift in the efficacy of neurotransmission in the LHb depending on the time of the recording. Our observations suggest that more careful examination is required to dissect the function of the LHb in future studies.

## Results

**Figure 1:**
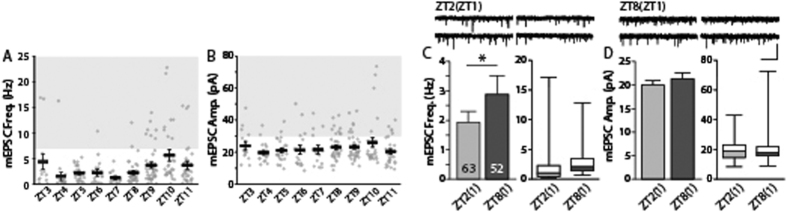
Frequency and amplitude of mEPSCs in the lateral habenula (LHb) are higher in the afternoon than in the morning. (**A**) mEPSCs frequencies obtained during ZT3–12 showed a significant variations depending on the time of recording (F(8, 219) = 3.46, p < 0.001 by one-way ANOVA test). The dominance of *high frequency* (>7 Hz) cell was increased in ZT8–12 than ZT3–7 (p < 0.05, χ^2^ test). (**B**) The mean mEPSCs amplitude per each hour during ZT3–12 remained comparable across the time point of recordings (F(8, 219) = 1.05, p > 0.3 by one-way ANOVA test). The dominance of *large amplitude* (>30 pA) cell was increased in ZT8–12 than ZT3–7 (p < 0.05, χ^2^ test). (**C**) mEPSC frequency was 1.87 ± 0.34 Hz (63 cells from 11 animals) during ZT2–6 (sacrificed at ZT1; ZT2(ZT1) group) and 2.88 ± 0.34 Hz (52 cells from 8 animals) during ZT8–12 (sacrificed at ZT1; ZT8(ZT1) group) (p < 0.05 by t-test, p < 0.05 by bootstrap). The box-plot shows the range of mEPSC frequencies obtained in each group. (**D**) The average mEPSC amplitude of the ZT2(ZT1) group was 20.14 ± 0.97 pA and the average of the ZT8(ZT1) group was 21.45 ± 1.63 pA (p > 0.47 by t-test, p > 0.4 by bootstrap). The box-plot shows the range of mEPSC amplitudes obtained. Representative traces are shown at the top of each panel (scale bars: 1 s and 40 pA).

When we plotted mEPSC frequencies and amplitudes against the time of recording from a large pool of accumulated recordings done in the coronal lateral habenula (LHb) slices of male Sprague Dawley rats during Zeitgeber time (ZT)3 (10:00) – ZT12 (19:00), we observed unexpected differences in mEPSC frequencies depending on the time of recording (Fig. 1A, F(8, 219) = 3.46, p < 0.001) but not in mEPSC amplitudes (Fig. 1B, F(8, 219) = 1.05, p > 0.3). Thus, we systemically investigated whether there are any temporal variations in basal synaptic transmission in the LHb. A previous study showed that PER2::luciferase (LUC) fusion protein photon signals peaks at ZT 12^12^,^13^. We, therefore, prepared acute brain slices at ZT1 and collected a substantial number of spontaneous excitatory transmission recordings from LHb neurons during ZT2 (9:00) – ZT6 (13:00) and ZT8 (15:00) – ZT12 (19:00). Interestingly, the frequency of mEPSCs was higher during ZT8–12 (ZT8(ZT1) group) compared to the frequency during ZT2–6 (ZT2(ZT1) group, Fig. 1C, p < 0.05 by t-test, p < 0.05 by bootstrap). However, mEPSC amplitudes of the ZT8(ZT1) group were not significantly different from those of the ZT2(ZT1) group (Fig. 1D, p > 0.4 by t-test, p > 0.5 by bootstrap).

**Figure 2:**
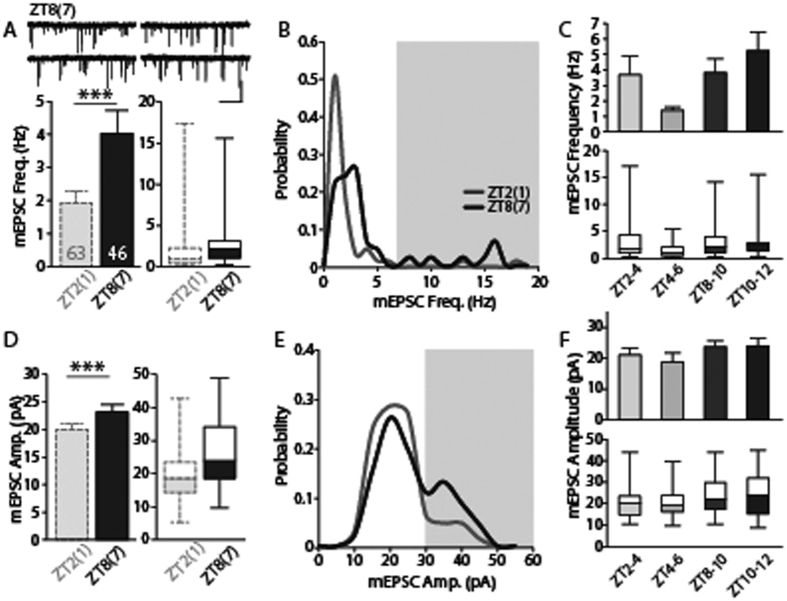
There are significant differences in the frequency and amplitude of mEPSCs of the lateral habenula (LHb) depending on the time of the day. (**A**) mEPSC frequency was 3.76 ± 0.65 Hz (46 cells from 11 animals) during ZT8–12 (sacrificed at ZT7; ZT8(ZT7) group), which was higher than that of the ZT2(ZT1) group (p < 0.01 by t-test, p < 0.01 by bootstrap) and comparable to that of the ZT8(ZT1) group shown in Fig. 1 (p > 0.2 by t-test, p > 0.2 by bootstrap). Representative traces are shown at the top of each panel (scale bars: 1 s and 40 pA). (**B**) mEPSC frequencies in each group were not normally distributed (ZT2(ZT1): p < 0.001; ZT8(ZT7): p < 0.001, Shapiro-Wilk test). In addition, the mEPSC frequency distribution at ZT8(ZT7) group was shifted to the right compared to that of ZT2(ZT1) group. There were significantly more neurons with *high-frequency* mEPSCs (>7 Hz) in the ZT8(ZT7) group (p < 0.05, χ^2^ test). (**C**) Analysis of mEPSC frequencies in 2-hr time window revealed a temporal oscillation during the light phase (F (3, 86) = 3.29, p < 0.05, one-way ANOVA), however there was no difference within ZT2(ZT1) group or ZT8(ZT7) group (p > 0.2 between ZT2-4 and ZT4-6; p > 0.9 between ZT8-10 and ZT10-12, Tukey’s post hoc test). (**D**) The average mEPSC amplitude in the ZT8(ZT7) group was 23.41 ± 1.34 pA, which was larger than the average amplitude in the ZT2(ZT1) group (p < 0.05 by t-test, p < 0.05 by bootstrap). (**E**) The mEPSC amplitudes in the two groups were not normally distributed (ZT2(ZT1): p < 0.001; ZT8(ZT7): p = 0.098, Shapiro-Wilk test) and the distribution was shifted towards larger amplitudes (>30 pA) in the ZT8(ZT7) group compared to the ZT2(ZT1) group (p < 0.05, χ^2^ test). Representative traces are shown at the top of the figure. (**F**) 2-hr time block analysis of mEPSC amplitudes showed no variations during light phase (F (3, 86) = 4.06, p > 0.4 one-way ANOVA).

To minimize any technical interference due to the long delay (>6 hours) between slice preparation and recording for the ZT8(ZT1) group, and to match the qualities of the recordings obtained during between different time windows, we prepared acute brain slices at ZT1 and ZT7, and then recorded during ZT2–6 and ZT8–12, respectively. Consistent with previous observations, the frequency of mEPSCs was higher during ZT8–12 (ZT8(ZT7) group) compared to the frequency during ZT2–6 (ZT2(ZT1) group) (Fig. 2A, p < 0.01 by t-test, p < 0.01 by bootstrap). When the same dataset was re-analyzed for narrower time windows (2-hr time blocks), there was a significant difference in mEPSC frequencies between recording time windows (Fig. 2C, F(3, 86) = 3.29, p < 0.05, one-way ANOVA), however there was no difference within defined groups (p > 0.2 between ZT2-4 and ZT4-6; p > 0.9 between ZT8-10 and ZT10-12, Tukey’s *post hoc* test). Notably, we observed a bimodal distribution of mEPSC frequencies in randomly chosen LHb neurons in both groups (Fig. 2B, p < 0.001, Shapiro-Wilk test), which is in agreement with our previous observations in VTA-projecting LHb neurons^21^. This observation suggests that the heterogeneity of synaptic transmission observed in the LHb is the physiological characteristics of this particular brain area which may not be driven or affected by circadian changes. The threshold for *high-frequency* mEPSCs was about 7–8 Hz, as reported previously^21^. However, the overall distribution of mEPSC frequencies was shifted toward the right in the ZT8 groups compared to the ZT2(ZT1) group, favoring the appearance of *high-frequency* cells in the afternoon. In addition, the frequency of mEPSCs in one out of the total of 41 recorded neurons in the ZT2(ZT1) group was higher than 7 Hz, while 8 out of the total of 45 LHb neurons showed *high-frequency* (>7 Hz) in the ZT8(ZT7) group. The dominance of neurons with *high-frequency* mEPSCs in the ZT8(ZT7) group was statistically significant, supporting that mEPSC frequencies are different depending on the time of the day (p < 0.05, χ^2^ test, Fig. 2B, shaded area). Interestingly, similar observations were made with our bulk recording reanalysis for each hour. The dominance of *high-frequency* mEPSCs was temporarily varied during the light phase of daily cycle (Fig. 1A, p < 0.05, χ^2^ test).

The mEPSC amplitudes in the ZT8(ZT7) group were increased compared to those of the ZT2(ZT1) group (Fig. 2D, p < 0.05 by t-test, p < 0.05 by bootstrap), although two-hr time block analysis revealed no significant difference in mEPSC amplitudes between time windows (Fig. 2F, F(3, 86) = 0.94, p > 0.4). In addition, the mEPSC amplitudes did not follow a normal distribution in either group (Fig. 2E, p < 0.05, Shapiro-Wilk test). Neurons with *large-amplitude* mEPSCs (>30 pA) were significantly more abundant in the ZT8(ZT7) group (7 out of 56 cells in the ZT2(ZT1) group *vs.* 12 out of 34 cells in the ZT8(ZT7) group, p < 0.05, χ^2^ test, Fig. 2E, shaded area). However, bulk recording reanalysis of the *large-amplitude* dominance in every hour did not reach statistical significance (Fig. 1B, p > 0.5, χ^2^ test). We found no statistical differences in recordings obtained during ZT8–12 in brain slices prepared at ZT1 and ZT7 (frequency: p > 0.2, amplitude: p > 0.3). This observation suggests that the time of sacrifice did not entrain the intrinsic clock of the LHb. Therefore, intrinsic temporal variations in mEPSC frequencies and amplitudes are maintained *in vitro* for at least 12 hours.

**Figure 3:**
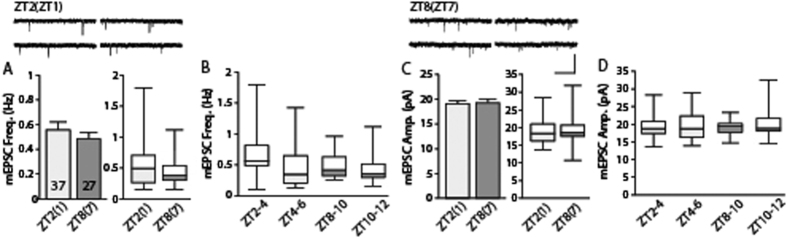
Frequency and amplitude of mEPSCs measured in the hippocampal CA1 region remain comparable during the light phase. (**A**) Frequencies of mEPSCs in the hippocampus were comparable between the ZT2(ZT1) group and the ZT8(ZT7) group (p > 0.40). The average mEPSC frequency was 0.53 ± 0.06 Hz in the ZT2(ZT1) group (37 cells from 5 animals) and 0.46 ± 0.05 Hz in the ZT8(ZT7) group (27 cells from 6 animals). (**C**) Amplitudes of mEPSC in the hippocampal CA1 region were comparable between groups (p > 0.84). The average mEPSC amplitude was 18.40 ± 0.62 pA in the ZT2(ZT1) group and 18.72 ± 0.78 pA in the ZT8(ZT7) group. Representative traces are shown at the top of each panel (scale bars: 1 s and 40 pA). (**B**,**D**) Distributions of mEPSC frequencies and amplitudes in every two hours are shown. There is no significant difference between time windows.

Next, to determine whether temporal variations in spontaneous transmission commonly occurs in other brain areas known to be circadianly controlled, we performed timely controlled recordings of spontaneous excitatory transmission from the hippocampal neurons as done in the LHb. There was no significant difference in both frequencies and amplitudes of mEPSCs between ZT2(ZT1) group and ZT8(ZT7) group (Fig. 3A,C, frequency: p > 0.4; amplitude: p > 0.8). When analyzed for 2-hr time blocks, we failed to observe any temporal differences between different time windows of recording (Fig. 3B,D, frequency: F(3, 62) = 1.28, p > 0.2; amplitude: F(3, 62) = 0.16, p > 0.9). These observations strongly suggest that temporal variations in the spontaneous transmission during the light phase are unique characteristics for LHb neurons.

**Figure 4:**
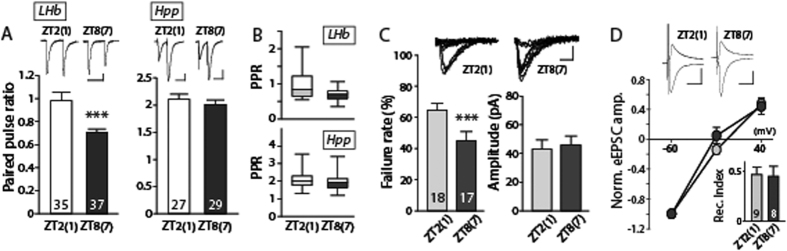
Presynaptic release probability in the lateral habenula changes depending on the time of the day. (**A**) Paired pulse ratios (PPRs, 2^nd^ eEPSC amplitude/1^st^ eEPSC amplitude) in the LHb were decreased in the ZT8(ZT7) group *vs.* the ZT2(ZT1) group (p < 0.01). The average PPR was 0.99 ± 0.06 (35 cells from 13 animals) in the LHb of the ZT2(ZT1) group and 0.67 ± 0.03 (37 cells from 17 animals) in the ZT8(ZT7) group. PPRs measured in the hippocampus (Hpp) remained comparable between the ZT2(ZT1) and ZT7 groups (p > 0.4). The mean PPR measured in the hippocampus of the ZT2(ZT1) group was 2.07 ± 0.10 (27 cells from 8 animals) *vs.* 1.97 ± 0.09 in the ZT8(ZT7) group (29 cells from 4 animals) (scale bar: 50 ms and 50 pA). (**B**) The box-plots show the ranges of the measured PPRs in the LHb and the hippocampus. (**C**) The failure rate of eEPSCs upon minimal stimulation in the LHb was 64.8 ± 4.1% in the ZT2(ZT1) group (18 cells from 5 animals) and 44.9 ± 5.9% in the ZT8(ZT7) group (17 cells from 7 animals). The failure rate was significantly lower in the ZT8(ZT7) group compared to the ZT2(ZT1) group (p < 0.01) (scale bar: 10 ms and 5 pA). (**D**) The rectification index (RI, eEPSC amplitude at 40 mV/eEPSC amplitude at −60 mV) of the LHb remained unchanged over time (p > 0.8, n = 8–9). The eEPSC amplitude at each holding potential was normalized to the eEPSC amplitude at −60 mV (scale bar: 20 ms and 100 pA). Representative traces are shown at the top of each panel.

The probability that an action potential invading the presynaptic terminal leads to successful neurotransmitter release varies depending on the specific synapses^22^ and can be modified under a variety of circumstances^23^. Changes in mEPSC frequencies often suggest changes in release probability^24^,^25^. Thus, we measured the presynaptic release probability of LHb neurons or hippocampal neurons by giving a pair of consecutive stimulations (50-ms apart) at different times of day. The reduced amplitude of evoked excitatory postsynaptic currents (eEPSCs) upon the second stimulation compared to the first stimulation often suggests the shortage of available synaptic vesicles due to insufficient time for vesicle recycling^23^. The reduction in the amplitude in response to the second stimulation is considered to be greater in synapses with higher release probability^23^. The paired pulse ratios (PPRs) of eEPSCs in the ZT8(ZT7) group were significantly lower than those of the ZT2(ZT1) group (Fig. 4A,B, p < 0.001). This observation, together with our observations regarding mEPSC frequencies, suggests that the presynaptic release probability is increased in the LHb in the ZT8(ZT7) group compared to the ZT2(ZT1) group. We failed to observe any significant differences in the PPRs measured in the hippocampus between the two groups (Fig. 4A,B, p > 0.4). We also examined the failure rate upon minimal stimulation intended to activate only few synapses to address changes in presynaptic efficacy. The failure rate was ~44% in the ZT8(ZT7) group and ~64% in the ZT2(ZT1) group, providing further support for our observation of increased release probability in the afternoon compared to the morning (Fig. 4C, p < 0.01). The average amplitude of the successful evoked events was comparable in both groups (Fig. 4D, p > 0.7).

To examine whether there are any alterations in the relative expressions of postsynaptic AMPA receptors with different permeabilities, we measured the rectification index in the presence of spermine in the pipette solution. We found no significant differences between the two groups (Fig. 4D, p > 0.8), suggesting that the oscillations in synaptic transmission may be primarily encoded presynaptically. Taken together, our observations strongly suggest that there are temporal variations in presynaptic release probability in the LHb during the light phase.

## Discussion

In this study, we showed that there are temporal variations in the efficacy of synaptic transmission occurring in the LHb. The synaptic efficacy in the LHb seems to be heterogeneous, resulting in bimodal distribution of mEPSC frequencies. Two populations of neurons are observed in both recording time windows however, the relative size of two populations seems to differ depending on the time windows of recording across the light phase. Interestingly, the rhythmicity seems mainly presynaptic: the frequency of mEPSCs and the release probability were increased in the afternoon compared to the morning while the relative expression of Ca^2+^ -permeable AMPA receptors remained comparable. This oscillation was maintained *ex vivo* for up to 12 hours.

The LHb is a highly heterogeneous structure, in which physiological properties of nearby neurons are not always comparable^21^,^26^,^27^,^28^,^29^. Our current observations add further complexity to the examination of the LHb. Randomized collection of data of substantially large size is not likely to skew the observation. However, if synaptic transmissions are recorded under two different conditions during separate time windows for the majority of each of the experiments, the observed differences could be due to intrinsic temporal variations and not the conditions or treatments of interest. Thus, we urge others to be more careful when analyzing synaptic transmission in the LHb in future studies.

Even though the heterogeneity *per se* in synaptic transmission occurring in the LHb is not likely under the temporal control, the relative occurrence of high activity neurons seems to be temporally controlled. Current observations cannot pinpoint the origin of temporal control whether it is governed by circadian changes in direct or indirect inputs including from the SCN or by oscillations in expression of clock proteins within the LHb. Given that we observed presynaptic variations as shown in changes in mEPSC frequencies and PPR, it is possible that circadian oscillations in input areas of the LHb may mediate temporal changes in neurotransmission onto the LHb. However, we failed to observe any temporal variations in synaptic transmission occurring in the hippocampus (Fig. 3), another area which is also known to be regulated by SCN^16^,^24^,^30^,^31^,^32^ and the excitability and synaptic plasticity in which are reported to be under the circadian control^33^. Some studies reported that temporal variations in the habenula complex was limited to the LHb^13^,^20^ however, recently, the medial habenular neurons are also shown to exhibit daily variations similar to our observations. In this study, daily variations were no longer observed in clock gene deficient animals, suggesting that the temporal dynamics could be directly regulated by the expression of clock-related genes^34^. Thus, the excitability of the entire habenula complex is likely under the circadian control.

Previous studies using organotypic LHb slice cultures or *in vivo* preparations also showed that there is a circadian oscillation in spontaneous firing rates in the LHb. Our current study in *ex vivo* acute brain slice preparation showed that neurotransmission onto LHb neurons is also temporally variable. More importantly, the variations include a significant shift in the dominance of highly active population of neurons and primarily rise from presynaptic efficacy of neurotransmitter release. This is of particular interest given that the LHb activity in animal models of depression were presynaptically but not postsynaptically potentiated and the high-frequency neurons are likely to make greater contribution to the observed abnormal potentiation in helpless rodent models^34^.

Currently available literatures suggest that the temporal variations in synaptic transmission and excitability are likely originated from the intrinsic circadian drive onto LHb neurons. Blockade of neuronal activity by tetrodotoxin (TTX) treatment failed to alter the circadian variations observed in the LHb^12^. The temporal variations in firing rates in the LHb^12^ and the mHb^34^ were not observed in animals lacking core circadian clock genes. What then may be the underlying cellular mechanisms mediating temporal variations in release probability in LHb synapses? One possible candidate is prokineticin 2, one of important circadian messenger which is released from the SCN and govern behavioral circadian rhythm^35^. Both mRNA and proteins for prokineticin 2 receptros are highly expressed in the LHb^35^,^36^,^37^ and prokineticin 2 is shown to alter the presynaptic release probability of GABA in the LHb^12^. In the hypothalamic paraventricular nucleus^38^, prokineticin 2 application increases the frequency of EPSCs in subpopulation of neurons. Therefore, the temporal oscillations in prokineticin release onto LHb neurons may mediate the observed variations in synaptic transmission. However, in SCN brain slices^39^ and in trigeminal ganglion primary cultures^40^, prokineticin 2 is reported to suppress GABA-mediated currents postsynaptically. Therefore future studies are required to examine the effect of prokineticin on the temporal variations in the LHb.

Alternatively, the voltage-gated Ca^2+^ channel (VGCC), which has been shown to regulate presynaptic vesicle release may play a role in temporal variations observed in the LHb. Several types of VGCCs such as the P/Q- and T-types are known to show clear circadian patterns of expression level changes in SCN neurons^1^,^41^. In addition, rhythmicity of Ca^2+^ influx in SCN neurons was maintained even in the presence of TTX^42^,^43^. The circadian oscillations of the Per1 and Per2 proteins are blocked by the inhibition of VGCCs^44^, suggesting that oscillatory Ca^2+^ flux, in combination with the rhythmic expression of VGCCs, are necessary for generating endogenous circadian oscillations and mediating rhythmic synaptic transmission. Therefore, Ca^2+^ oscillation mediated by the VGCCs in LHb neurons may contribute to the generation of diurnal differences in synaptic transmission in the LHb.

A number of studies suggest that circadian rhythmicity is important, primarily for physiologically anticipatory activities^45^,^46^,^47^,^48^. Disruption in circadian rhythms often accompanies other clinical conditions. One shared symptom observed in patients with schizophrenia and depression is the presence of aberrant circadian rhythms^49^,^50^, including insomnia^3^,^50^. Several studies employing different stressors observed impaired circadian rhythms of molecular clock proteins including Per1, Clock, and Cry2 in the amygdala, the limbic forebrain and the hypothalamus^51^,^52^. These studies suggest that stress can disrupt circadian rhythms of brain areas related to emotional processing. Recently, circadian disruption with shorter lengths of the daily cycle has been shown to directly lead to depressive behaviours in rodents in the absence of other stressors through the action of melanopsin-expressing neurons^53^. The LHb is known to project to major monoamine centres such as the VTA, the dorsal raphe and the locus coeruleus. Previously, we have shown that mEPSCs from VTA-projecting LHb neurons are presynaptically potentiated in animal models of depression^21^. Temporal variations in the release probabilities of LHb neurons may thus determine the strengths of the synaptic inputs to monoamine centres, thereby differentially regulating the release of monoamines. Therefore, it is likely that presynaptic temporal variations in neurotransmission of the LHb are altered in different emotional contexts, such as in addicted or stressed conditions. Future studies are anticipated to elucidate molecular mechanisms underlying the rhythmicity of neurotransmission in the LHb and possible alterations in response to different emotional stimuli.

## Materials and Methods

### Animals

All procedures were carried out in accordance with the guidelines of the National Institutes of Health for animal care and use (http://oacu.od.nih.gov/regs/index.htm) and approved by the Institutional Animal Care and Use Committee of the Konkuk University (KU12063 and KU14155, Seoul, Korea). Male Sprague-Dawley rats were purchased from the Orient Bio company (a branch of Charles River, Gapyung, Korea) and group-housed with free access to food and water under standard conditions. Animals remained in a climate- and light-controlled environment (22 ± 1 °C, 45% humidity, 12:12-hour light/dark cycle with lights on at 7 am) for at least a week before the experiments. Zeitgeber time (ZT) 0 was defined as lights-on (07:00) and ZT12 was defined as lights-off (19:00).

### Slice preparation

Animals (6–8 weeks old) were anaesthetized using isoflurane. After immediate decapitation, brains were stored in ice-cold dissection buffer (in mM: 212 sucrose, 3 KCl, 26 NaHCO_3_, 1.25 NaH_2_PO_4_, 7 MgCl_2_, and 10 glucose, gassed with 95% O_2_ and 5% CO_2_). Transverse slices (350-μm thick) containing the hippocampus and the LHb were prepared using a Leica VT 1000 S Vibratome. Brain slices were transferred to a recovery chamber containing artificial cerebrospinal fluid (aCSF) (in mM, 118 NaCl, 2.5 KCl, 11 glucose, 1 NaH_2_PO_4_, and 26.2 NaHCO_3_, gassed with 95% O_2_ and 5% CO_2_) at 35 °C for one hour and then stored at room temperature. Slice preparation was performed at ZT1 (08:00) and ZT7 (14:00). All experiments were performed at 27–30 °C.

### Electrophysiology

CA1 pyramidal neurons or LHb neurons (mostly in the medial part of the LHb) were voltage-clamped to −60 mV in warmed aCSF using Axopatch 200B and Clampex 10.3 (Molecular Devices), or HEKA EPC8 and pulse v8.8 (HEKA Electronik), filtered at 5 kHz and sampled at 10 kHz. Glass pipettes with a resistance of 2–6 MΩ were filled with an internal solution containing the following (in mM): 115 Cs methanesulphonate, 20 CsCl, 10 HEPES, 2.5 MgCl_2_, 0.6 EGTA, 5 QX314, 4 Na_2_-ATP, 0.4 Na_2_-GTP, and 10 Na-phosphocreatine (pH 7.3). For recordings of evoked transmission, QX314 (5 mM) was added to the internal solution. The Schaffer collateral pathway (in the hippocampus) or the stria medullaris (in the LHb) was stimulated to elicit eEPSCs using platinum/iridium cluster electrodes. Each response was recorded for 15 sweeps with 20 sec intervals to average the amplitude of eEPSCs. The ionotropic GABAergic receptor antagonist picrotoxin (PTX, 50 μM; Sigma, in DMSO) was added to the aCSF to exclude GABAR-mediated inhibitory synaptic transmission. mEPSCs were recorded in the presence of 1 μM TTX and 50 μM PTX, and analysed manually to avoid false-positive and false-negative events using Mini Analysis software (Synaptosoft).

### Data analysis

Data were analyzed using Clampfit 10.3 (Molecular Devices) or Mini Analysis software (Synaptosoft). Values are presented as means ± standard error of the mean (SEM). *n* indicates the number of cells studied. Shapiro-Wilk test was used for testing normality for data sets. Two-tailed unpaired *t*-tests, bootstraps for mean and, one-way ANOVA were used for statistical comparisons between groups unless stated otherwise. For bootstrapping which relies on random sampling with replacement to improve the comparison between not-normally distributed data sets, means (N_i_ and M_i_) of two data sets (N and M of size n and m) which were randomly sampled n and m times, respectively were calculated. The generation of means was repeated 10,000 times. If N_j_ was more than M_j_ fewer than 1 or 5% of the times, then the probability that N is more than M was considered to be less than 0.01 or 0.05, respectively.

## Additional Information

**How to cite this article**: Park, H. *et al*. Temporal variations in presynaptic release probability in the lateral habenula. *Sci. Rep.* **7**, 40866; doi: 10.1038/srep40866 (2017).

**Publisher's note:** Springer Nature remains neutral with regard to jurisdictional claims in published maps and institutional affiliations.
